# Gender and Sexual Orientation Differences in Sexist Attitudes Among Korean Adults: A MIMIC Model Approach

**DOI:** 10.3390/bs16020207

**Published:** 2026-01-30

**Authors:** Minsun Lee, Hyun-Hwa Lee

**Affiliations:** 1 Department of Fashion Design, Konkuk University, Glocal Campus, Chungju 27478, Republic of Korea; minsunlee@kku.ac.kr; 2 Department of Fashion Design & Textiles, Inha University, Incheon 22212, Republic of Korea

**Keywords:** ambivalent sexism theory, sexism, non-heterosexual, mimic model, Korean

## Abstract

The ambivalent sexism theory supports differences in the manifestations of sexism among individuals with diverse genders and sexual orientations. However, it still remained unclear whether individuals who share common strong cultural values endorse different levels of sexism according to their gender and sexual orientation. The current study aimed to examine differences in sexist attitudes based on gender and sexual orientation among Korean adults. We first tested measurement invariance in a Korean Multi-dimensional Sexism Inventory (K-MSI) between heterosexuals (*n* = 374) and sexual minorities (*n* = 445), and second, we compared the latent means across groups using the Multiple Indicators Multiple Causes (MIMIC) model. The results confirmed the first-order six-factor structure of the K-MSI with adequate internal consistency, and supported partial scalar invariance across heterosexual and sexual minority men and women. The MIMIC model approach revealed significant age, gender, and sexual orientation differences in most of hostile sexism (HS) and benevolent sexism (BS) components. Overall, heterosexuals reported higher levels of sexism than non-heterosexuals within each gender. Gender differences in BS have become nuanced when sexual orientation was considered. The current study also provides an overview of Korean culture that may uniquely influences individuals’ sexist attitudes, which would interest international researchers.

## 1. Introduction

Sexism refers to prejudice or discrimination based on sex and/or gender, mainly used to describe discrimination against women (Glick & Fiske, 2001). Ambivalent sexism theory assumes that heteronormativity and gendered power relations shape attitudes toward men and women. This theory also posits that sexism consists of both hostile sexism (HS), which reflects overtly negative attitudes, and benevolent sexism (BS), which reflects seemingly positive but are actually harmful attitudes toward women. Due to the deeply rooted gender stereotypes in society, men and women tend to endorse different levels of sexist beliefs. Previous studies have reported that men generally show higher levels of HS than women, while findings regarding gender differences in BS have been inconsistent (Davies, 2004; Glick et al., 2000). Additionally, the emphasis on heterosexual intimacy within the theory implies that sexual minorities may perceive sexism differently from heterosexuals. Cowie et al. (2019) argued that sexual minority individuals who develop sexist attitudes based on sociocultural influences express similar levels of sexism as heterosexuals, while those with a strong sense of personal identity express less sexism than heterosexuals.

Individuals within the same cultural contexts share common social norms and cultural values that may affect their beliefs regarding sex and gender roles. Cultural factors, along with gender and sexual orientation, may contribute to sexism (Etengoff & Lefevor, 2021). Especially in Korea, sexism has historically been highly prevalent, and various deeply rooted social norms and cultural values, such as Confucianism, Protestantism, and patriarchy, have influenced perceptions of gender roles (Jung, 2021; Kim & Park, 2018). These cultural values have reinforced gender and sexual prejudices, which are closely linked to sexist beliefs and behaviors. Despite the fact that many Asian countries hold distinctly different cultural values from Western countries and even within Asia, with each country having its unique social and cultural norms (Sinha, 1996), studies related to sexism have been relatively Western-oriented. However, it still remained unclear whether individuals who share strong cultural values, but have different genders and sexual orientations (i.e., heterosexual and non-heterosexual Korean men and women), exhibit different levels and patterns in their sexist beliefs. To address this gap, the current study employs the Korean Multi-dimensional Sexism Inventory (K-MSI), which was developed to capture the multifaceted nature of sexism within the Korean sociocultural context. Unlike other widely used sexism measures, the K-MSI reflects culture-specific features of Korean society that shape perceptions of gender roles and discrimination. Investigating how sexism manifests across gender and sexual orientation within the Korean context would expand theoretical understanding and address a significant gap in the existing literature.

### 1.1. Ambivalent Sexism Theory

The ambivalent sexism theory posits that sexism toward men and women exhibits ambivalent properties, comprising a mixture of antipathy and subjective benevolence (Glick & Fiske, 1996, 1999). Regarding sexist beliefs about women, the theory explains that men’s attitudes toward women entail not only disparagement but also protection and admiration. This theory positions these ambivalent sexist attitudes as Hostile Sexism (HS) and Benevolent Sexism (BS). Specifically, HS is defined as overtly hostile attitudes toward women who violate traditional norms for gender roles, while BS refers to interrelated protective attitudes toward women who fulfil traditional gender roles (Glick & Fiske, 1996, 2001). HS characterizes women as inferior to men, while BS considers women as dependent on men and idealizes women in traditional roles. Even though BS involves subjectively positive feelings toward women (i.e., women should be cherished and protected by men), it is still based on gender stereotypes and perceptions of women’s lower status. Furthermore, Glick and Fiske (1996) explained that both HS and BS contain three components: paternalism, gender differentiation, and heterosexuality, each of which manifests differently in hostile and benevolent forms of sexism. Specifically, paternalism appears as dominative paternalism in HS (HS-P) and protective paternalism in BS (BS-P); gender differentiation as competitive gender differentiation in HS (HS-G) and complementary gender differentiation in BS (BS-G); and heterosexuality as heterosexual hostility in HS (HS-H) and heterosexual intimacy in BS (BS-I).

Even though these conceptual explanations seem to distinguish between HS and BS, they are positively correlated. From the male perspective, BS, which is socially acceptable, is necessary to neutralize hostile sexism, which is socially discouraged (Chisango et al., 2015). In Chisango et al.’s study, heterosexual, married, Black African women reported that their husbands were likely to display hostile sexist behaviors in private contexts while showing benevolent sexist behaviors more often in public contexts. Glick and Fiske (1996) also argued that sexism across cultures has continued due to the coexistence of HS and BS. Glick et al. (2000) compared the levels of HS and BS among a sample of 15,000 men and women across 19 countries. Their results supported that the ambivalent conceptual structure of sexism exists across cultures and that HS and BS are coherent constructs across countries, with BS acting as a complement to HS.

### 1.2. Ambivalent Sexism Across Gender and Sexual Orientation

Individuals form and develop sexist attitudes under the influence of personal identities (i.e., age, gender, and sexual orientation) and socio-cultural environments (Cowie et al., 2019; Gaunt, 2012). In general, women tend to report lower levels of hostile sexism (HS) than men across ages, countries, and cultures (i.e., Davies, 2004 for English college students; García-Senlle et al., 2024 for Spanish adolescents; Garaigordobil & Aliri, 2013 for a Spanish community sample; Lee & Cunningham, 2016 for American college students; Sakalli, 2002 for Turkish college students; Tekkas et al., 2020 for Korean nursing students). On the other hand, gender differences in BS have shown inconsistent patterns across ages and countries. For example, Glick et al. (2000) reported that women were more likely to accept BS than men in countries where gender inequality and overall sexism were high, such as Cuba, Nigeria, South Africa, Botswana, and Colombia. On the other hand, studies reported that men were more likely to have greater benevolent sexism (BS) than women in the samples from the U.S., Korea, Australia, England, and the Netherlands (Ahn et al., 2005; Glick & Fiske, 1996; Glick et al., 2000).

Sexual minorities may perceive the concepts of ambivalent sexism, especially their focus on heterosexual intimacy, differently from heterosexuals (Cross et al., 2021). The ambivalent sexism theory also supports differences in the manifestation of HS and BS across heterosexual and non-heterosexual men and women, since traditional binary gender norms and heteronormativity are crucial factors in the perpetuation of sexist attitudes and behaviors (Cowie et al., 2019). According to the ambivalent sexism theory (Glick & Fiske, 1996), heterosexuality is one of the most powerful sources of sexism. Since this may not be applicable to non-heterosexuals whose attractions are often toward same-sex individuals, non-heterosexuals are less likely to be concerned about sexism toward men and women as compared to heterosexuals. However, while several studies have explored gender differences in sexism, research on differences in sexist attitudes depending on individuals’ sexual orientation is scarce. Additionally, most existing studies have adopted the Ambivalent Sexism Inventory (ASI; Glick & Fiske, 1996), which was developed within Western cultural contexts. A recent study comparing the observed mean scores of sexism as assessed by the ASI, across gay/lesbian, bisexual, and heterosexual men and women in national samples of New Zealanders, reported significant differences in HS and BS based on gender and sexual orientation (Cowie et al., 2019). In their study, heterosexuals were found to endorse both HS and BS significantly more so than lesbian/gay and bisexual participants, regardless of gender. Heterosexual men reported significantly higher mean scores of HS and BS than heterosexual women. However, heterosexual women reported higher scores in BS than gay and bisexual men. Although these findings provide important insights into how sexism varies across gender and sexual orientation, they also highlight the need to examine how sexism manifests in other cultural contexts.

### 1.3. Cultural Values and Sexism in Korea

The social norms and cultural values in Korea, such as Confucianism, patriarchy, collectivism, conservative religions (i.e., Protestantism, Buddhism, and Catholicism), and mandatory military service, emphasize traditional gender roles and heteronormativity, and thus influence individuals’ attitudes toward each gender and towards sexual minorities (Rich et al., 2021). As a moral and philosophical ideology, Confucianism is a dominant influence on social norms in Korea, shaping the lifestyles of Koreans in various social contexts, including family relations, career development, politics, and education (Hyun, 2001; Soh, 1993). Confucian philosophy emphasizes harmonious relationships and appropriateness within a society, which is believed to be attained when people fulfill traditional gender roles (Gao et al., 2012). In Confucian societies, a man whose roles are son, husband, and/or father should lead his family, while a woman should play the roles of daughter, wife, and/or mother by taking care of her family (Park & Schepp, 2015). Thus, Confucianism strictly separates the roles of men and women, emphasizing a gender hierarchy that views women as subordinate. In line with this notion, Confucianism is closely associated with patriarchy, a social system in which men hold primary authority within the family as leaders and decision-makers, while women are often excluded from these roles (Cho, 1996). Several studies have found that Confucian patriarchy is deeply ingrained into every aspect of Koreans’ lives, promoting gender inequality and sexist attitudes, beliefs, and behaviors (Cho, 1996; Oh & Oh, 2022). By providing men with power over women, both Confucianism and patriarchy reinforce sexism toward women. Moreover, since many collectivistic societies tend to be more hierarchical and highly patriarchal, members of those societies may be more likely to be overtly sexist. Within collectivistic societies emphasizing interconnectedness within groups and the importance of harmonious relationships (Triandis, 2001), people are primarily concerned with maintaining relationships with others and prioritize group goals over personal goals. Related to sexism, the cultural value of collectivism has promoted gendered attitudes by emphasizing women’s duties and obligations to foster harmonious relationships within families and societies (Davis & Williamson, 2019). Thus, all of these cultural values in Korea impose gender stereotypes of masculinity and femininity, which guide how individuals act based on their sex.

Another critical source for expectations around gender roles is religion (Mikołajczak & Pietrzak, 2014). Ever since Protestantism was first introduced into Korea, it has steadily grown and is now the largest religious group (19.73%) in the country followed by Buddhism (15.53%) and Catholicism (7.93%); yet individuals with no religious affiliation (56.06%) still constitute the largest group (Korean Statistical Information Service, 2017). Especially in Korea, Protestantism, Buddhism, and Catholicism have all been influenced by Confucian and patriarchal social values–hierarchical structures, such as authority based on age and sex (Min & Kim, 2005), which has led to strong conservative tendencies in Korean religions (Ok, 2011; So, 2019). Moreover, these religions are particularly opposed to homosexuality. The Christian Council of Korea has publicly stated that homosexuality is contrary to the teachings of the Bible. The Korean council of Religious Leaders also released a statement opposing the push for the enactment of a law prohibiting discrimination against homosexuals, based on the belief that they undermine traditional values and social norms (Cha, 2016).

Mandatory military service for males can also contribute to societies’ overall sexist attitudes. As of April 2023, all able-bodied Korean males have to complete compulsory military service for an 18-month period. One of the dominant beliefs in military culture is that masculinity is ideal and superior to femininity (Sasson-Levy, 2003). Heteronormative gender norms and hypermasculinity in the military context can often create biased attitudes towards women, as well as towards homosexual individuals (Archer, 2013; Poulin et al., 2018). One of the few existing studies by Uğurlu and Özdemir (2017) reported that attitudes fostered by the masculine structure of the military were positively associated with both HS and BS after controlling for sex in a Turkish sample. Some studies have also described that the military conscription system and male-dominated culture in the military may result in gender- and sexual orientation-based discrimination (Gibbons & Rossi, 2022; Gitzen, 2022; Han et al., 2017).

Research has repeatedly demonstrated that conservative attitudes are closely linked to binary gender norms (Prusaczyk & Hodson, 2020) and more negative attitudes toward homosexuality (Sakalli, 2002). The gender-related social norms emphasized in Confucianism, patriarchy, collectivism, conservative religions, and mandatory military service may create sexist attitudes, as well as gender and sexual inequality in Korea. Overall, these social norms and cultural values in Korea align with the major elements of sexism by reinforcing ideas of paternalism (men are more powerful than women), gender differentiation (men and women should behave in line with traditional gender roles), and heterosexuality (men should only have close, intimate relationships with women).

### 1.4. The Current Study and Research Hypotheses

The current study aimed to examine differences in sexism across gender and sexual orientation among Korean adults. We first evaluated the factor structure and measurement invariance of the Korean Multi-dimensional Sexism Inventory (K-MSI) among Korean heterosexual and non-heterosexual men and women. Then, we examined the latent mean differences in sexism across groups using the Multiple Indicators Multiple Causes (MIMIC) model. Establishing measurement invariance is particularly important for the measure of ambivalent sexism among sexual orientation groups since sexism is guided by norms to which sexual minorities do not conform, such as binary gender norms and heterosexuality (Cross et al., 2021).

Based on the aforementioned literature review, we hypothesized that men would endorse greater sexism in all components than women (H1) and that heterosexuals would endorse greater sexism in all components than non-heterosexuals (H2). In addition, since the BS-I subcomponent is based on the concept of heterosexual intimacy, non-heterosexual individuals would be less concerned with opposite-sex romantic relationships. Thus, we hypothesized that non-heterosexual men would endorse similar levels of BS-I as non-heterosexual women (H3), and be less likely to endorse BS-I as compared to heterosexual women (H4).

## 2. Materials and Methods

### 2.1. Participants and Procedures

We collected data from a total of 850 Korean adults (400 heterosexuals and 450 non-heterosexuals) using an online questionnaire. Prior to the analyses, outliers were screened and excluded following standard recommendations for multivariate data analysis (Tabachnick & Fidell, 2007). After excluding 31 responses containing outliers, the remaining data from 819 respondents contained no missing data, and were thus used for data analysis. The final sample comprised 374 heterosexuals (181 men and 193 women) and 445 non-heterosexuals (189 men and 256 women). Participants’ ages ranged from 20 to 64 years (*M* = 43.86 years, *SD* = 13.27) for heterosexuals, and from 20 to 56 years (*M* = 27.12 years, *SD* = 5.18) for non-heterosexuals.

We recruited heterosexual participants from a marketing research firm, while recruiting non-heterosexual participants through various social media channels and online communities for sexual minorities. We provided information about the study along with an informed consent form. Only those who agreed to voluntarily participate proceeded to the main questionnaire. Upon completion, participants received compensation for their participation. Ethics approval was granted by the University’s Institutional Review Board.

### 2.2. Measures

We assessed sexism using the Korean Multi-dimensional Sexism Inventory (K-MSI; Korean Women’s Development Institute, 2007), which contains 24 items with 12 items for each of HS and BS subscales. For the HS subscale, the K-MSI contains three subcomponents of Dominative Paternalism (P), Competitive Gender Role Differentiation (G), and Heterosexual Hostility (H), while the BS subscale is further categorized into three subcomponents: Protective Paternalism (P), Complementary Gender Differentiation (G), and Heterosexual Intimacy (I). HS-P measures the belief that men are inherently superior to women and therefore entitled to dominance; HS-G assesses the belief that inherent gender differences justify men’s superiority in socially valued roles; and HS-H measures the belief that women use sexual attraction and heterosexual intimacy to exert control over men. In contrast, BS-P assesses the belief that women should be protected by men due to their perceived vulnerability, BS-G captures the belief that men and women possess different but complementary traits suited to distinct social roles, and BS-I measures the belief that romantic heterosexual relationships are essential to happiness and that women should embody warmth and sexual desirability. Items corresponding to each subcomponent are provided in Appendix A. Participants responded to items on a scale ranging from 1 (*disagree strongly*) to 4 (*agree strongly*). Higher scores indicated greater perceived HS and BS. In the present study, the values of ꞷ for the six subcomponents ranged from 0.72 to 0.92 for heterosexuals and from 0.76 to 0.93 for non-heterosexuals.

Participants provided their background demographics, including age, gender identity (cisgender men and women, genderqueer/gender non-conforming, trans male/men and trans female/woman, and different identity), sex assigned at birth (female or male), sexual orientation, and education. We assessed sexual orientation using the Klein Sexual Orientation Grid (KSOG; Klein et al., 1985). Participants selected either 1 (=heterosexual only), 2 (=heterosexual mostly), 3 (=heterosexual somewhat more), 4 (=hetero/gay-lesbian/queer/non-hetero equally), 5 (=gay-lesbian/queer/non-hetero somewhat more), 6 (=gay-lesbian/queer/non-hetero mostly), or 7 (=gay-lesbian/queer/non-hetero only). Following previous studies (Soulliard & Vander Wal, 2019), we considered participants with responses of 1 through 3 to be heterosexual, while participants whose responses ranged from 4 through 7 were categorized as non-heterosexuals.

### 2.3. Statistical Analysis Plan

We computed descriptive statistics, omega values for internal consistency of the scale, and correlations among variables using IBM SPSS Statistics for Windows, Version 27.0 (IBM Corp., Armonk, NY, USA). We tested confirmatory factor analysis (CFA) for the total sample as well as for each of the four heterosexual and non-heterosexual men and women samples separately. After an adequate model fit was established, we examined measurement invariance with multigroup confirmatory factor analysis (MGCFA) across the four groups by testing successively restrictive levels, including configural, metric, and scalar measurement invariance. If the results did not confirm full invariance across groups, we consulted the modification indices to determine which item residuals we should unconstrain across groups to establish partial invariance. We evaluated goodness-of-fit for the measurement model based on various statistical indices, such as the Satorra–Bentler scaled chi square (S-Bχ^2^), the Root Mean Square Error of Approximation (RMSEA), Comparative Fit Index (CFI), and Standardized Root Mean Square Residual (SRMR). Following the recommendation by Hu and Bentler (1999), we considered values close to 0.06 and about 0.07–0.08 for the RMSEA and CFI values close to 0.95 and 0.90 to be a good and adequate fit, respectively, and SRMR values close to 0.08 to be a good fit. We evaluated measurement invariance based on the following criteria suggested by Chen (2007): a non-significant change in the χ^2^ value at *p* < 0.05, RMSEA differences of 0.015 or less, CFI differences of 0.01 or less, and SRMR differences of 0.030 or less indicated a lack of measurement variance.

After establishing (partial) scalar invariance, we proceeded with the Multiple Indicators Multiple Causes (MIMIC) model testing to examine the differences in sexism across groups. To test the mean comparisons, it is essential to confirm whether the scales would provide comparable measurements for all individuals and groups. Researchers can meaningfully compare scores across groups only when the scores are equivalently measured (Bauer, 2017), i.e., measurement invariance should be established to test differences in scores across groups. Researchers should establish at least partial scalar invariance to compare means across groups (Davidov et al., 2012). In the current study, we examined a series of MIMIC models in the total sample, each heterosexual and non-heterosexual sample, and a combined sample of heterosexual women and non-heterosexual men. The MIMIC model allows researchers to test the associations between the latent variables and covariates. We used age, gender (0 = men and 1 = women), and sexual orientation (0 = non-heterosexuals and 1 = heterosexuals) as covariates. The influence of these three covariates on six subcomponents of sexism was estimated simultaneously via standardized path coefficients. We considered significant regression effects of covariates on the latent variable as indicating latent mean differences. We conducted measurement model and MIMIC model testing using M*plus* 8.8.

## 3. Results

### 3.1. Preliminary Analysis

We present descriptive statistics for the six subcomponents of the K-MSI, correlations among variables, and the values of ꞷ for each subcomponent for heterosexuals and non-heterosexuals across sex in Table 1 and Table 2, respectively. All six subcomponents of the K-MSI were positively intercorrelated for all four groups. This indicates that different forms of sexism are systematically related while remaining conceptually distinct. This results also align with the ambivalent sexism theory, which posits that hostile and benevolent forms of sexism tend to co-occur across gender and sexual orientation groups.

### 3.2. CFA

The initial first-order six-factor model of the K-MSI showed an adequate fit to the data in the total sample, as well as in each group of heterosexual and non-heterosexual men and women. We present fit indices for each model in Table 3. In all models, the standardized estimated parameters of all items were significant, ranging from 0.73 to 0.92, and the AVE values were 0.52 or higher for all six subcomponents (≥0.50), confirming the convergent validity of the measurement model.

### 3.3. Measurement Invariance

We present the results of MGCFA across all four groups including the values of the fit indices for each model and model comparisons in Table 4. The configural invariance model provided a good fit to the data. The metric invariance was supported (∆RMSEA = 0.001, ∆CFI = 0.007, and ∆SRMR = 0.009), while the scalar invariance was not established because the change in CFI exceeded the recommended threshold of 0.01 (∆RMSEA = 0.003, ∆CFI = 0.014, and ∆SRMR = 0.002). Thus, we tested the partial scalar invariance after we freed the intercepts of items 4 and 7 in the non-heterosexual women group based on the modification indices. This modified model showed a good fit, and its comparison to the metric invariance model supported partial scalar invariance across the groups (∆RMSEA = <0.001, ∆CFI = 0.005, and ∆SRMR = 0.005).

### 3.4. MIMIC Model and Latent Mean Differences

Table 5 presents the path coefficients for the associations between covariates and the six subcomponents of sexism in the MIMIC models in the total sample, each heterosexual and non-heterosexual sample, and a combined sample of heterosexual women and non-heterosexual men. In the total sample, the MIMIC model with the three covariates of age, gender, and sexual orientation revealed a good fit, S-Bχ^2^ = 1113.97, df = 291, *p* < 0.001, RMSEA = 0.056, CFI = 0.936, SRMR = 0.041. The addition of the three covariates significantly affected the loadings of all six subcomponents of sexism. The path coefficients for the effects of age on the six subcomponents of sexism were significant and positive (β ranged from 0.233 to 0.378, all *ps* < 0.001), suggesting that older participants tended to have more sexist attitudes than younger participants. The path coefficients for the effects of gender on the six sexism constructs were significant and negative (β ranged from −0.153 to −0.372, all *ps* < 0.001), indicating that women were more likely to show less sexism than men. Thus, H1 was supported. The associations between sexual orientation and the six sexism constructs were also significant and positive (β ranged from 0.181 to 0.404, all *ps* < 0.001, except for BS-P showing *p* < 0.01). This demonstrates that heterosexual participants showed more sexism than non-heterosexuals, supporting H2.

In the heterosexual sample, the results showed significantly positive path coefficients between age and the six subcomponents of sexism (β ranged from 0.258 to 0.433, all *p*s < 0.001). All path coefficients for the gender effects on the six subcomponents of sexism were significant and negative (β ranged from −0.392 to −0.199, all *p*s < 0.001). These results indicate that older heterosexual participants were more likely to endorse sexism than their younger counterparts, while heterosexual women were less likely to endorse sexism than heterosexual men.

In the non-heterosexual sample, the path coefficients from age to the four subcomponents of sexism including HS-P, HS-H, BS-P, and BS-I, were significant and positive (HS-P: β = 0.098, *p* < 0.05, HS-H: β = 0.158, *p* < 0.001, BS-P: β = 0.114, *p* < 0.05, and BS-I: β = 0.155, *p* < 0.05). The older non-heterosexual participants were more likely to show greater sexist attitudes in these four subcomponents. On the other hand, all path coefficients from gender to the sexism subcomponents were significant and negative (β ranged from −0.423 to −0.318, all *p*s < 0.001), except BS-I (β = −0.044, *p* = 0.382). Non-heterosexual men were more likely to endorse all the subcomponents of HS, BS-P, and BS-G. However, there was no significant gender difference in BS-I, supporting H3.

In a combined sample of heterosexual women and non-heterosexual men, the path coefficients from age to all six subcomponents of sexism were significant and positive (β ranged from 0.336 to 0.421, all *p*s < 0.001). The path coefficients from gender to the three HS subcomponents were significant and negative (β ranged from −0.257 to −0.199, all *p*s < 0.001). Gender had a significant and negative effect on BS-P (β = −0.171, *p* < 0.01), while revealing a non-significant effect on BS-G (β = 0.075, *p* = 0.200) and a significant and positive effect on BS-I (β = 0.200, *p* < 0.001). Thus, H4 was supported.

## 4. Discussion

The current study examined the differences in sexist attitudes across heterosexual and non-heterosexual men and women within the Korea context. Since the K-MSI was previously validated only in heterosexual dominant samples, we first validated its factor structure in non-heterosexual samples and tested measurement invariance across the gender and sexual orientation groups. After confirming the partial scalar invariance of the K-MSI across heterosexual and non-heterosexual men and women, we examined the latent mean differences across groups using the MIMIC model.

Our results supported the construct validity of the K-MSI across diverse genders and sexual orientations. Consistent with previous studies validating the K-MSI with heterosexuals (Korean Women’s Development Institute, 2007), a first-order six-factor structure of the scale was confirmed in non-heterosexual men and women. Furthermore, the measurement invariance analyses supported partial scalar invariance across heterosexual and non-heterosexual men and women after freely estimating the intercepts of items 4 and 7 in the non-heterosexual women group. This result may have been due to the fact that items 4 and 7 contained statements with somewhat strong language (i.e., *unconditionally*) and information regarding military service required only for men. As both gender inequality and mandatory military service for men are both issues which have received a great deal of attention in Korea, Korean women may be particularly sensitive to these issues and thus these items may have elicited strong reactions. These results may also reflect the fact that Korea’s mandatory conscription system uniquely influences gendered expectations and sexist attitudes, thereby underscoring the importance of sociocultural factors specific to the Korean context.

The results of the MIMIC model tests provided important implications for differences in sexism according to gender and sexual orientation. Despite the results that all three factors—age, gender, and sexual orientation—had significant associations with the six sexism subcomponents in the total sample, further analyses of each sample of heterosexual and non-heterosexual individuals revealed distinct patterns of associations among the covariates and latent variables of sexism. Consistent with previous studies (Lee & Cunningham, 2016; Sakalli, 2002; Tekkas et al., 2020), men were more likely to endorse HS than women, even when comparing heterosexual women and non-heterosexual men. These results may explain that men are likely to maintain their privilege in various social contexts by endorsing HS beliefs (Cowie et al., 2019). Additionally, long-standing cultural values, such as Confucian patriarchy and conservative religions, may contribute to the higher levels of HS among heterosexual men as they emphasize traditional gendered norms and hierarchical gender relations. At the same time, it is important to acknowledge that certain Confucian values (i.e., emphasizing diligence and family responsibility) have been reinterpreted under contemporary Korean society, potentially contributing to changes in gender norms. On the other hand, heterosexual women were more likely to endorse BS-I as compared to non-heterosexual men. This may reflect an adaptive response to dominant gender norms. For heterosexual women, rejecting benevolent sexism could be perceived as involving social disadvantages within a patriarchal and heteronormative society. It is important to note that, although BS frames gender inequality in a subjectively positive manner, it still remains sexist insofar as it reinforces traditional gender roles despite its positive framing. In the non-heterosexual sample, gender differences in BS-I were not significant. The items in BS-I contain the concept of heterosexual intimacy which were established from the perspective of heteronormativity. Non-heterosexual individuals might not be interested in these issues because they differ from the sexual attractions they feel (Cowie et al., 2019). These results align with the ambivalent sexism theory, which explains that sexism is maintained through society’s gender norms and heteronormativity (Glick & Fiske, 1996). The lower endorsement of BS-I among non-heterosexual individuals implies the theoretical importance of heterosexuality in the manifestation of ambivalent sexism. Moreover, the absence of gender differences in BS-I among non-heterosexual individuals may also be related to shared experiences of social disadvantages and power imbalances, which could lead these individuals to hold similar attitudes toward sexism.

By adopting the MIMIC model, the results of the latent mean comparisons of this study can provide more accurate results than the composite mean score comparisons. However, we should discuss the age differences in levels of sexism that were found mainly in the heterosexual sample in the current study. This can result from the sample characteristics in that our heterosexual sample was diversely distributed across a broad age range while the non-heterosexual sample mostly comprised younger individuals. In addition, the positive associations between the age and sexism subcomponents should be carefully interpreted because our MIMIC model assumed linear relationships between covariates and latent variables. A handful of studies on the differences in sexism across age have reported U-shape trajectories for sexism across the life span, except for benevolent sexism in men (Garaigordobil & Aliri, 2013; Hammond et al., 2018).

The correlation analysis results supported Glick and Fiske’s (1996) ambivalent structure of sexism, which proposed that hostile sexism can coexist with benevolent sexism. In all of the samples of heterosexual and non-heterosexual men and women used in this study, all six subcomponents of the K-MSI showed moderate-to-large intercorrelations, supporting and extending previous studies across cultures (Glick & Fiske, 1996; Glick et al., 2000). In the Korean context, individuals who endorse high levels of derogatory attitudes toward women may be more likely to endorse paternalistic and patronizing attitudes toward women regardless of their gender and sexual orientation. In addition, based on Cohen’s (1992) conventions of effect size interpretations (correlation values of 0.10, 0.30, and 0.50 indicating weak, moderate, and strong associations), the intercorrelations among the three subcomponents of HS indicated strong associations, whereas those among the BS subcomponents revealed moderate associations in all four groups of heterosexual and non-heterosexual men and women. This might be explained by the fact that conservative ideology is more closely linked to HS than BS (Austin & Jackson, 2019).

### Limitations and Future Research Directions

First, despite the fact that we discussed several sociocultural values affecting sexism in the Korean context, the current study did not actually assess individuals’ perceptions of those values nor did it examine the associations between cultural values and sexism. Further research investigating how various unique social and cultural values in Korea (e.g., attitudes toward military service, patriarchy, or religious conservatism) influence individuals’ sexist attitudes would provide additional support for the rationales discussed in this study. Second, the results of this study, including measurement invariance across gender and sexual majority and minority groups, and latent mean differences in sexism across these groups, can be interpreted only within the Korean context. Despite these valuable insights regarding sexism across cultures, future studies should investigate differences in sexism based on gender and sexual orientation within a cross-cultural perspective. Third, we should consider sexual fluidity, which means flexibility in sexual orientation. An individual’s sexual attractions, identity, and behavior can change across one’s lifespan, which is especially common among sexual minority individuals (Kinnish et al., 2005). As most of the non-heterosexual participants in this study were in their 20s to early 30s, it is highly likely that they could change their sexual identity in the future. In addition, with the same reasoning, the results of this study can be only interpreted for individuals within these age ranges. Future research tracing and comparing sexism over time would extend our knowledge on this topic. Last, given that sexism is a complex concept influenced by various factors, we recommend that future studies incorporate more diverse personal, social, and cultural factors in identifying the differences in sexism across gender and sexual orientation. Moreover, as the current study supports the metric and partial scalar measurement invariance of the K-MSI across heterosexual and non-heterosexual Korean men and women, further research could compare the structural associations among the related variables across sexual majority and minority individuals.

## 5. Conclusions

The findings of the current study support the notions that ambivalent sexism exists across cultures, that hostile and benevolent sexism are intercorrelated, and that these sexist attitudes differ according to age, gender, and sexual orientation across cultures (Cowie et al., 2019; Glick & Fiske, 1996; Glick et al., 2000). Moreover, to fully comprehend levels of sexism across genders and sexual orientations, it is necessary to understand the social norms and cultural values shared not only within each gender and sexual orientation-related community but also in the country as a whole. The current study provides an overview of the particularities of Korean culture that can lead to sexist attitudes. This contribution could attract international researchers by providing valuable information to help understand how cultural, gender, and sexual orientation influences sexist attitudes in Korea. The results of this study can also provide insights into the ways in which gender and sexual orientation are intertwined with attitudes towards sexism, as well as shed light on potential disparities in sexism across different gender and sexual orientation groups.

## Figures and Tables

**Table 1:** Descriptive statistics and correlations among study variables for heterosexuals.

	Women (*n* = 193)	Men (*n* = 181)	Correlations							
Variables	*M* (*SD*)	*M* (*SD*)	1	2	3	4	5	6	7	8
1. HS-P	1.62 (0.59)	2.14 (0.63)	--	0.86 ***	0.72 ***	0.46 ***	0.51 ***	0.57 ***	0.36 ***	−0.14
2. HS-G	1.62 (0.60)	2.17 (0.62)	0.82 ***	--	0.74 ***	0.46 ***	0.49 ***	0.55 ***	0.36 ***	−0.08
3. HS-H	1.77 (0.66)	2.28 (0.72)	0.67 ***	0.69 ***	--	0.38 ***	0.42 ***	0.43 ***	0.37 ***	−0.08
4. BS-P	1.95 (0.62)	2.39 (0.64)	0.20 **	0.20 **	0.18 *	--	0.54 ***	0.54 ***	0.38 ***	−0.06
5. BS-G	2.46 (0.72)	2.74 (0.58)	0.33 ***	0.30 ***	0.32 ***	0.42 ***	--	0.67 ***	0.44 ***	−0.22 **
6. BS-I	1.98 (0.79)	2.47 (0.74)	0.24 **	0.23 **	0.28 ***	0.28 ***	0.46 ***	--	0.37 ***	−0.23 **
7. Age	43.40 (13.03)	44.35 (13.54)	0.15 *	0.11	0.21 **	0.46 ***	0.32 ***	0.14	--	−0.22 **
8. Education level	-	-	−0.09	−0.09	−0.12	−0.06	−0.17 *	−0.14	<0.00	--
ꞷ for women [95% CI]	--	--	0.85 [0.80, 0.89]	0.88 [0.85, 0.91]	0.89 [0.85, 0.92]	0.85 [0.80, 0.89]	0.84 [0.80, 0.88]	0.92 [0.90, 0.94]	--	--
ꞷ for men [95% CI]	--	--	0.81 [0.76, 0.86]	0.84 [0.79, 0.88]	0.87 [0.82, 0.90]	0.84 [0.78, 0.88]	0.72 [0.59, 0.81]	0.88 [0.84, 0.91]	--	--

Note. HS-P = Hostile sexism-dominative paternalism, HS-G = Hostile sexism-competitive gender differentiation, HS-H = Hostile sexism-heterosexual hostility, BS-P = Benevolent sexism-protective paternalism, BS-G = Benevolent sexism-complementary gender differentiation, BS-I = Benevolent sexism-heterosexual intimacy. Results for women are reported in the upper diagonal area. * *p* < 0.05. ** *p* < 0.01. *** *p* < 0.001.

**Table 2:** Descriptive statistics and correlations among study variables for non-heterosexuals.

	Women (*n* = 256)	Men (*n* = 189)	Correlations							
Variables	*M* (*SD*)	*M* (*SD*)	1	2	3	4	5	6	7	8
1. HS-P	1.15 (0.35)	1.63 (0.64)	--	0.79 ***	0.68 ***	0.24 ***	0.42 ***	0.30 ***	−0.01	−0.17 **
2. HS-G	1.15 (0.38)	1.66 (0.70)	0.83 ***	--	0.78 ***	0.25 ***	0.42 ***	0.24 ***	−0.03	−0.15 *
3. HS-H	1.15 (0.43)	1.82 (0.85)	0.78 ***	0.74 ***	--	0.18 **	0.32 ***	0.29 ***	0.05	−0.15 *
4. BS-P	1.49 (0.56)	1.90 (0.68)	0.27 ***	0.25 **	0.23 **	--	0.33 ***	0.37 ***	0.06	−0.04
5. BS-G	1.56 (0.58)	2.02 (0.71)	0.61 ***	0.61 ***	0.59 ***	0.36 ***	--	0.34 ***	−0.06	−0.13 *
6. BS-I	1.26 (0.54)	1.36 (0.61)	0.50 ***	0.44 ***	0.51 ***	0.34 ***	0.48 ***	--	0.13 *	−0.02
7. Age	25.77 (4.48)	28.96 (5.50)	0.16 *	0.12	0.24 **	0.14	0.16 *	0.18 *	--	0.17 *
8. Education level	--	--	−0.08	−0.13	−0.10	−0.02	−0.03	−0.14	0.15 *	--
ꞷ for women [95% CI]	--	--	0.79 [0.67, 0.87]	0.86 [0.78, 0.91]	0.91 [0.87, 0.95]	0.81 [0.75, 0.85]	0.76 [0.68, 0.82]	0.87 [0.79, 0.92]	--	--
ꞷ for men [95% CI]	--	--	0.83 [0.77, 0.87]	0.88 [0.83, 0.90]	0.93 [0.91, 0.95]	0.85 [0.80, 0.89]	0.83 [0.78, 0.88]	0.91 [0.87, 0.95]	--	--

Note. HS-P = Hostile sexism-dominative paternalism, HS-G = Hostile sexism-competitive gender differentiation, HS-H = Hostile sexism-heterosexual hostility, BS-P = Benevolent sexism-protective paternalism, BS-G = Benevolent sexism-complementary gender differentiation, BS-I = Benevolent sexism-heterosexual intimacy. Results for women are reported in the upper diagonal area. * *p* < 0.05. ** *p* < 0.01. *** *p* < 0.001.

**Table 3:** Goodness-of fit indices for six-factor model of the K-MSI.

Group	S-B*χ*^2^	RMSEA	CFI	SRMR
***Each of heterosexual and non-heterosexual men and women groups***				
Heterosexual men	442.43_(237)_	0.069	0.898	0.084
Heterosexual women	460.04_(237)_	0.070	0.922	0.056
Non-heterosexual men	467.65_(237)_	0.072	0.908	0.064
Non-heterosexual women	394.85_(237)_	0.051	0.915	0.071
***Combined groups***				
Heterosexuals	615.39_(237)_	0.065	0.925	0.059
Non-heterosexuals	561.96_(237)_	0.056	0.931	0.053
All samples	920.71_(237)_	0.059	0.941	0.042

**Table 4:** Measurement invariance across heterosexual and non-heterosexual men and women.

Model	S-B*χ*^2^	RMSEA	CFI	SRMR	Reference Model	∆S-B*χ*^2^	∆RMSEA	∆CFI	∆SRMR	*p*
**MGCFA**										
Configural	1748.68_(948)_	0.064	0.912	0.069						
Metric	1863.37_(1002)_	0.065	0.905	0.078	Configural	112.86_(54)_	0.001	0.007	0.009	<0.001
Scalar	2043.64_(1056)_	0.068	0.891	0.080	Metric	198.39_(54)_	0.003	0.014	0.002	<0.001
Partial scalar (item 4 & 7 in non-heterosexual women)	1961.61_(1054)_	0.065	0.900	0.073	Metric	98.69_(52)_	<0.001	0.005	0.005	<0.001

**Table 5:** MIMIC model results.

Subcomponent	Covariates	Total Sample		Heterosexuals		Non-Heterosexuals		Hetero Women and Non-Hetero Men	
		*β*	S.E.	*β*	S.E.	*β*	S.E.	*β*	S.E.
HS-P	Age Gender Sexual orientation	0.246 *** −0.363 *** 0.229 ***	0.042 0.029 0.040	0.262 *** −0.392 *** --	0.045 0.042 --	0.098 * −0.419 *** --	0.050 0.043 --	0.359 *** −0.199 *** --	0.062 0.056 --
HS-G	Age Gender Sexual orientation	0.233 *** −0.357 *** 0.208 ***	0.039 0.029 0.038	0.258 *** −0.390 *** --	0.042 0.042 --	0.071 −0.390 *** --	0.045 0.042 --	0.336 *** −0.212 *** --	0.056 0.050 --
HS-H	Age Gender Sexual orientation	0.276 *** −0.372 *** 0.181 ***	0.037 0.029 0.038	0.287 *** −0.362 *** --	0.039 0.042 --	0.158 *** −0.423 *** --	0.044 0.041 --	0.367 *** −0.257 *** --	0.051 0.052 --
BS-P	Age Gender Sexual orientation	0.378 *** −0.293 *** 0.120 **	0.040 0.029 0.041	0.433 *** −0.335 *** --	0.045 0.039 --	0.114 * −0.318 *** --	0.049 0.047 --	0.373 *** −0.171 ** --	0.060 0.061 --
BS-G	Age Gender Sexual orientation	0.305 *** −0.232 *** 0.358 ***	0.038 0.028 0.039	0.417 *** −0.199 *** --	0.047 0.045 --	0.070 −0.368 *** --	0.053 0.047 --	0.421 *** 0.075 --	0.057 0.059 --
BS-I	Age Gender Sexual orientation	0.241 *** −0.153 *** 0.404 ***	0.040 0.028 0.039	0.267 *** −0.307 *** --	0.047 0.043 --	0.155 * −0.044 --	0.053 0.051 --	0.370 *** 0.200 *** --	0.056 0.053 --

Note. * *p* < 0.05. ** *p* < 0.01. *** *p* < 0.001.

## Data Availability

The data that support the findings of this study are available from the corresponding author upon reasonable request.

## Appendix A

**Table A1:** Item list of the Korean Multi-dimensional Sexism Inventory (K-MSI).

K-MSI
***Hostile sexism-dominative paternalism (HS-P)*** 1. Having a responsible male student as the class president, rather than a female student, leads to better class management. 2. Women frequently whine despite the fact that men should naturally lead the world. 3. Men should be given more decision-making power than women as there are so many things to do for our society. 4. Women demand rights without having to fulfill responsibilities such as military service. ***Hostile sexism-competitive gender differentiation (HS-G)*** 5. Women are prone to emotional instability, making it difficult to entrust them with significant tasks. 6. Women tend to get upset more easily than men, leading to less rational handling of tasks. 7. Women demand absolute equality unconditionally, while ignoring the disparities between men and women. 8. Women lack decisiveness when compared to men, making it difficult for them to perform important roles. ***Hostile sexism-heterosexual hostility (HS_H)*** 9. Women only seek out men when they face difficulties or need favors. 10. Women utilize their beauty to manipulate men. 11. Women prefer pricey luxury items and expect men to pay for all dating costs. 12. Women initiate sexual advances and, if they are not satisfied, claim sexual harassment. ***Benevolent sexism-protective paternalism (BS-P)*** 13. Men should give way to women when they meet on a narrow walkway. 14. Men should bear the burden of physically hazardous tasks rather than women. 15. On cold days, men should offer their jackets to women. 16. Even though the load is light, men should carry it rather than allow women to. ***Benevolent sexism-complementary gender differentiation (BS-G)*** 17. When it comes to taking care of the household, women possess greater attention to detail than men. 18. Women are capable of raising children and managing household chores. 19. Having a mother at home to greet children after school benefits their emotional development. 20. Since men are intelligent and women are emotional, they achieve harmony when they engage in tasks that align with their respective strengths. ***Benevolent sexism-heterosexual intimacy (BS-I)*** 21. Men will never be completely happy unless they receive love from women. 22. Men can live a complete life when they have a loving woman in their lives. 23. A man who has earned a woman’s love can be considered a true man. 24. A man’s life would be meaningless without a loving lady.

Note. We provided an English version of the scale items for informational purposes only. Even though it was translated by professional translators, we did not evaluate the scale’s factor structure and psychometric properties among English-speaking individuals.
